# Relationship between personal care products usage and triclosan exposure: the second Korean National Environmental Health Survey (KoNEHS 2012–2014)

**DOI:** 10.1186/s40557-019-0283-y

**Published:** 2019-01-28

**Authors:** Minkyu Park, Seyoung Kim, Yeji Kim, Do Jin Nam, Jae-Hong Ryoo, Sinye Lim

**Affiliations:** 1 Department of Occupational and Environmental Medicine, Kyung Hee University Medical Center, Seoul, South Korea; 2 Department of Occupational and Environmental Medicine, School of Medicine Kyung Hee University, Seoul, Korea

**Keywords:** Triclosan, Personal care products, Korean National Environmental Health Survey

## Abstract

**Background:**

We aimed to find the exposure level of triclosan (TCS), a known endocrine disruptor, related to the use of personal care products using a nationally representative data of the general population in Korea.

**Methods:**

This study included data of 6288 adults aged 19 years and older (2692 men, 3596 women), based on the Second Korean National Environmental Health Survey (KoNEHS 2012–2014). The data were divided according to gender. The frequency and proportion of each variable were determined by dividing participants into two groups based on the top 75th percentile concentration of urinary TCS (male: 1.096 μg/g creatinine, female: 1.329 μg/g creatinine). Odds ratios (ORs) were calculated using logistic regression analysis for the high TCS exposure and low TCS exposure groups.

**Results:**

Overall, the proportion of participants using personal care products was higher in women than in men. There was a significantly higher proportion of participants in the high TCS exposure group with younger age, higher education and income levels and with more frequent use of fragrance products, hair care products, body cleansers, cosmetics, and antimicrobial agents. In both men and women, ORs tended to increase with increased frequency of use of hair care products, body cleansers, and cosmetics before and after adjustment.

**Conclusions:**

Our findings demonstrate that as the frequency of use of personal care products increases, urine TCS concentration increases. Because TCS is a well-known endocrine disruptor, further studies are needed and explore other health effects with exposure to TCS in general population in Korea.

**Electronic supplementary material:**

The online version of this article (10.1186/s40557-019-0283-y) contains supplementary material, which is available to authorized users.

## Background

Triclosan (TCS), chemical name 5-chloro-2-(2,4-dichlorophenoxy)phenol, is a synthetic antimicrobial and antifungal agent, which is widely used throughout the world. TCS is used mainly as a disinfectant in soaps, detergents, toothpaste, mouthwash, fabrics, deodorant, shampoo, and plastic additives, and it is used in many personal care products, animal products, and industry and household products [1].

TCS is absorbed into the body through the mouth and skin [2]. TCS is absorbed at a rate of nearly 100% in the oral cavity, with a half-life of 10–20 h; less than 10% is absorbed via the transdermal route, with a half-life of 1.4–2.1 days. Absorbed TCS is detected mainly in plasma, urine, and breast milk [3]; it is mostly excreted in the urine [4]. According to a pharmacokinetic study, after oral administration of 4 mg TCS, most was excreted in the urine within the first 24 h and levels had recovered to baseline within 8 days after administration [5]. These results demonstrate that urinary TCS concentrations can be used as a biomarker of TCS exposure [6]. There have been several studies indicating that urinary TCS concentrations are associated with the use of personal care products containing TCS [6–8].

TCS is known to be an endocrine disruptor that is structurally similar to polychlorinated biphenyls, bisphenol A, dioxins, and thyroid hormones [9]. TCS increases estradiol (E2) and progesterone secretion [10], has a similar effect as estrogen [11], and can induce hypothyroxinemia [12]. Moreover, in experiments with mice, rats, and hamsters, TCS has been associated with liver tumors [4]. Contact dermatitis, photosensitivity, contact dermatitis, skin irritation, and other conditions have also been reported in association with TCS [13, 14].

Because of these health problems, various regulations have recently been established in relation to TCS in many countries. Various studies on TCS exposure levels have also been carried out. However, studies on TCS levels in Korea and related factors have not yet been conducted. In this study, we used a nationally representative data for the general population in Korea to analyze the level of TCS exposure in the general population according to the frequency of use of personal care products, which are the main exposure source of TCS.

## Material and methods

### Study participants

This study was based on data of the Second Korean National Environmental Health Survey (KoNEHS 2012–2014). According to Article 14 of the Environmental Health Act, the KoNEHS is conducted every 3 years to investigate the human exposure levels of harmful environmental factors in the Korean population, explore the influencing factors, and continuously investigate the spatial and temporal distribution and change in these factors [15].

The survey area consisted of 16 cities and provinces nationwide. For the survey sample, the enumeration districts of the National Population and Housing Census 2010 were used for the sample population with the initial stratification classified by local administration. Secondary stratification was according to socioeconomic factors, using stratified multistage cluster sampling applied with proportional allocation of the square root of the population to extract 400 sample enumeration districts [16]. Approximately 15 people from each sample enumeration district were surveyed, targeting a total of 6478 Koreans aged 19 years and older. The survey consisted of an environmental exposure-related questionnaire, clinical testing, and analysis of harmful environmental substances in biological samples [15].

In this study, after excluding 190 participants with missing urinary TCS concentration data, 6288 participants (2692 men, 3596 women) were included from among a total of 6478 participants.

### Variables

#### Variables of interest

To determine the association between TCS exposure and frequency of personal care products use, the frequency of use was classified as used rarely, less than once a week, and more than once a week. Personal care products were classified as hair care products, body cleansers, make-up products, nail care products, antibacterial agents, and fragrance products. Hair care products referred to products such as waxes, gels, and sprays. Body cleansers included foam cleansers, body wash, and shampoo, but not soap. Make-up products included cosmetic products other than basic cosmetics, such as skin care products and lotions; sun block products were also included. Antimicrobial agents refer to liquid-type antibacterial or sterilizing products [17].

Sociodemographic variables and health behavior-related variables of participants were analyzed as follows. Age and body mass index (BMI) were averaged as sociodemographic variables. Education level was classified as below a middle school graduate level, high school graduate level, or college graduate and above. Marital status was classified as unmarried, married, or other (divorced, widowed, separated), and socioeconomic status was classified into quartiles according to household income. Participants were classified as never, former or current smokers and alcohol drinkers. Participants were also categorized according to physical activity level as regularly performing no exercise, moderate exercise, or vigorous exercise.

#### Urinary triclosan

Urinary TCS concentration was measured using ultra performance liquid chromatography – tandem mass spectrometry (UPLC–MS/MS) with the Xevo TQ-S (Waters Corporation, Milford, MA, USA) using a spot urine sample [18]. After hydrolyzing each urine sample with ß-glucuronidase/arylsulfatase-degrading enzyme, the metabolites of TCS were extracted with ethyl ether and measured. The measuring principle of TCS concentration was to apply a calibration curve prepared by the standard addition method with using standard solution to the sample. For the precision control in the whole analysis process, relative standard deviation (RSD) of two samples of low dose of standard solution should be less than 15% for within batch and 20% for between batch. Quality control (QC) sample was used for the accuracy control in the analysis process. The concentrations less than the method detection limit (MDL, 0.500 μg/L) were substituted with values of MDL divided by the square root of 2. The final TCS concentration for statistical analysis was calculated after adjusting urinary creatinine concentration.

### Statistical analysis

The frequency and proportion of each variable were presented after stratification by gender to determine the general characteristics of participants. First, participants were divided into a high TCS exposure group and low TCS exposure group, based on the top 75th percentile concentration of urinary TCS (male: 1.096 μg/g creatinine, female: 1.329 μg/g creatinine). The frequency and proportion of each variable were then determined by gender. Chi-square tests were performed to analyze the difference in the distribution of each variable. Odds ratios (ORs) and 95% confidence intervals (CIs) were calculated using logistic regression for the high TCS exposure and low TCS exposure groups. Unadjusted models are presented and a multivariate adjusted model was adjusted for age, BMI, socioeconomic variables, and health behavior-related variables. IBM SPSS version 19 for Windows (SPSS Inc., Chicago, IL, USA) was used for statistical analysis and the statistical significance level was set at *p* < 0.05.

## Results

The distribution of the independent variables in the present study was as follows (Table 1). A total 2692 (42.8%) participants were male, and 3596 (57.2%) were female. A greater percentage of men than women were college graduates or higher, and more men than women were current drinkers and smokers. In terms of fragrance products, hair care products, nail care products, antimicrobial agents, and air fresheners usage, more than half of men and women responded that they rarely used these products. More than half of men said they rarely used cosmetics and body cleansers, whereas more than half of women reported using these products once a week.

**Table 1:** Basic characteristics according to sociodemographic factors and several exposure factros to triclosan in study population

Category		Total (*n* = 6288)	Male (*n* = 2692)	Female (*n* = 3596)
Triclosan level (μg/L)		0.35 (0.35–1.21)	0.35 (0.35–1.09)	0.35 (0.35–1.33)
Age (years)		51.6 (15.1)	51.7 (15.5)	51.4 (14.8)
BMI (kg/m^2^)		24.3 (3.4)	24.6 (3.2)	24.1 (3.5)
Education	≤ Middle school	2207 (35.1)	762 (28.3)	1445 (40.2)
	High school	1901 (30.2)	822 (30.5)	1079 (30.0)
	≥ College	2180 (34.7)	1108 (41.2)	1072 (29.8)
Marital status	Single	632 (10.0)	346 (12.9)	286 (7.9)
	Married	4992 (79.4)	2213 (82.2)	2779 (77.3)
	Others	664 (10.6)	133 (4.9)	531 (14.8)
Household income	1st quartile	1767 (28.1)	769 (28.6)	998 (27.8)
	2nd quartile	2900 (46.1)	1261 (46.8)	1639 (45.6)
	3rd quartile	1563 (24.9)	634 (23.6)	929 (25.8)
	4th quartile	58 (0.9)	28 (1.0)	30 (0.8)
Smoking	None	4147 (66.0)	742 (27.6)	3405 (94.7)
	Ex-smoker	1028 (16.3)	964 (35.8)	64 (1.8)
	Current-smoker	1013 (17.7)	986 (36.6)	127 (3.5)
Alcohol	None	2153 (34.2)	429 (15.9)	1724 (47.9)
	Ex	412 (6.6)	274 (10.2)	138 (3.9)
	Current	3723 (59.2)	1989 (73.9)	1734 (48.2)
Exercise	No	3307 (52.6)	1309 (48.6)	1998 (55.6)
	Moderate	709 (11.3)	284 (10.6)	425 (11.8)
	Vigorous	2272 (36.1)	1099 (40.8)	1173 (32.6)
Fragrance products	Rarely	5023 (79.9)	2269 (84.3)	2754 (76.6)
	< Once a week	403 (6.4)	122 (4.5)	281 (7.8)
	≥ Once a week	862 (13.7)	301 (11.2)	561 (15.6)
Hair care products	Rarely	3838 (61.1)	1948 (72.4)	1890 (52.5)
	< Once a week	732 (11.6)	251 (9.3)	481 (13.4)
	≥ Once a week	1718 (27.3)	493 (18.3)	1225 (34.1)
Body cleansers	Rarely	2958 (47.0)	1902 (70.7)	1056 (29.4)
	< Once a week	110 (1.8)	46 (1.7)	64 (1.8)
	≥ Once a week	3220 (51.2)	744 (27.6)	2476 (68.8)
Cosmetics	Rarely	3154 (50.2)	2381 (88.5)	773 (21.5)
	< Once a week	252 (4.0)	81 (3.0)	171 (4.8)
	≥ Once a week	2882 (45.8)	230 (8.5)	2652 (73.7)
Nail care products	Rarely	5325 (84.7)	2681 (99.6)	2644 (73.5)
	< Once a week	678 (10.8)	8 (0.3)	670 (18.6)
	≥ Once a week	285 (4.5)	3 (0.1)	282 (7.9)
Antimicrobial agents	Rarely	5234 (83.2)	2320 (86.2)	2914 (81.0)
	< Once a week	213 (3.4)	57 (2.1)	156 (4.4)
	≥ Once a week	841 (13.4)	315 (11.7)	526 (14.6)
Air freshener	Rarely	4673 (74.3)	2017 (74.9)	2656 (73.9)
	< Once a week	231 (3.7)	73 (2.7)	158 (4.4)
	≥ Once a week	1384 (22.0)	602 (22.4)	782 (21.7)

Data are mean (standard deviation), median (interquartile range), or number (%)

Urine TCS concentration was determined according to high and low TCS exposure groups, based on the upper 75th percentile concentration; the distribution of independent variables is presented in Table 2. The average age in the high TCS exposure group was significantly lower than that in the low exposure group. Furthermore, the urinary TCS concentration tended to decrease as age increased (See Additional file 1: Table S1). For both genders, the proportions of college graduates and participants with household income in the third quartile or higher, were significantly greater in the high TCS exposure group than in the low exposure group; the proportions of current drinkers and participants who vigorously exercised were also higher in the high TCS exposure group. Distribution by frequency of use of personal care products showed that for both genders, perfumes, hair care products, body cleansers, cosmetics, and antimicrobial agents were used more frequently in the high TCS exposure group than in the low exposure group. Additionally, more women in the high TCS exposure group reported more frequent use of nail care products.

**Table 2:** Basic characteristics of study population in both high^a^ (the top 75th percentile) and low urinary concentration of triclosan group by gender

		Male			Female		
Category		Low (*n* = 2016)	High (*n* = 676)	*p* value	Low (*n* = 2696)	High (*n* = 900)	*p* value
Age (years)		52.6 (15.3)	49.0 (15.9)	< 0.0001	52.3 (14.7)	48.8 (14.7)	< 0.0001
BMI (kg/m^2^)		24.5 (3.1)	24.7 (3.3)	0.219	24.1 (3.5)	23.9 (3.5)	0.034
Education	≤ Middle school	618 (30.6)	144 (21.3)	< 0.0001	1162 (43.1)	283 (31.4)	< 0.0001
	High school	633 (31.4)	189 (28.0)		783 (29.0)	296 (32.9)	
	≥ College	765 (38.0)	343 (50.7)		751 (27.9)	321 (35.7)	
Marital status	Single	234 (11.6)	112 (16.6)	0.003	187 (6.9)	99 (11.0)	< 0.0001
	Married	1678 (83.2)	535 (79.1)		2088 (77.5)	691 (76.8)	
	Others	104 (5.2)	29 (4.3)		421 (15.6)	110 (12.2)	
Household income	1st quartile	598 (29.6)	171 (29.3)	0.039	790 (29.3)	208 (23.1)	< 0.0001
	2nd quartile	947 (47.0)	314 (46.4)		1223 (45.4)	416 (46.2)	
	3rd quartile	451 (22.4)	183 (27.1)		663 (24.6)	266 (29.6)	
	4th quartile	20 (1.0)	8 (1.2)		20 (0.7)	10 (1.1)	
Smoking	None	552 (27.4)	190 (28.2)	0.898	2551 (94.6)	854 (94.9)	0.135
	Ex-smoker	721 (35.8)	243 (35.9)		43 (1.6)	21 (2.3)	
	Current smoker	743 (36.8)	243 (35.9)		102 (3.8)	25 (2.8)	
Alcohol	None	333 (16.5)	96 (14.2)	< 0.0001	1323 (49.1)	401 (44.5)	0.036
	Ex	227 (11.3)	47 (6.9)		96 (3.6)	42 (4.7)	
	Current	1456 (72.2)	533 (78.9)		1277 (47.3)	457 (50.8)	
Exercise	No	1007 (49.9)	302 (44.7)	0.029	1538 (57.0)	460 (51.1)	0.005
	Moderate	215 (10.7)	69 (10.2)		300 (11.1)	125 (13.9)	
	Vigorous	794 (39.4)	305 (45.1)		858 (31.9)	315 (35.0)	
Fragrance products	Rarely	1740 (86.3)	529 (78.3)	< 0.0001	2106 (78.1)	648 (72.0)	< 0.0001
	< Once a week	83 (4.1)	39 (5.8)		192 (7.1)	89 (9.9)	
	≥ Once a week	193 (9.6)	108 (15.9)		398 (14.8)	163 (18.1)	
Hair care products	Rarely	1510 (74.9)	438 (64.8)	< 0.0001	1442 (53.5)	448 (49.8)	0.113
	< Once a week	179 (8.9)	72 (10.6)		360 (13.3)	121 (13.4)	
	≥ Once a week	327 (16.2)	166 (24.6)		894 (33.2)	331 (36.8)	
Body cleansers	Rarely	1496 (74.2)	406 (60.1)	< 0.0001	858 (31.8)	198 (22.0)	< 0.0001
	< Once a week	13 (1.6)	13 (1.9)		49 (1.8)	15 (1.7)	
	≥ Once a week	487 (24.2)	257 (38.0)		1789 (66.4)	687 (76.3)	
Cosmetics	Rarely	1814 (90.0)	567 (83.9)	< 0.0001	629 (23.3)	144 (16.0)	< 0.0001
	< Once a week	56 (2.8)	25 (3.7)		136 (5.0)	35 (3.9)	
	≥ Once a week	146 (7.2)	84 (12.4)		1931 (71.6)	721 (80.1)	
Nail care products	Rarely	2007 (99.5)	674 (99.7)	0.604	2024 (75.1)	620 (68.9)	0.001
	< Once a week	6 (0.3)	2 (0.3)		469 (17.4)	201 (22.3)	
	≥ Once a week	3 (0.2)	0 (0.0)		203 (7.5)	79 (8.8)	
Antimicrobial agents	Rarely	1763 (87.4)	557 (82.4)	0.004	2215 (82.2)	699 (77.7)	0.008
	< Once a week	38 (1.9)	19 (2.8)		114 (4.2)	42 (4.7)	
	≥ Once a week	215 (10.7)	100 (14.8)		367 (13.6)	159 (17.6)	
Air fresheners	Rarely	1517 (75.3)	500 (73.9)	0.55	2014 (74.7)	642 (71.3)	0.13
	< Once a week	57 (2.8)	16 (2.4)		113 (4.2)	45 (5.0)	
	≥ Once a week	442 (21.9)	160 (23.7)		569 (21.1)	213 (23.7)	

^a^ Male:≥ 1.096 μg/g creatinine, female: ≥1.329 μg/g creatinine, data are mean (standard deviation), or number (%)

Logistic regression analysis was applied to determine the degree of association between the frequency of personal care products use and urinary TCS concentration according to low TCS and high TCS exposure groups (Table 3). In the case of men, fragrance products, hair care products, body cleansers, and cosmetics showed significantly higher ORs for use more than once a week versus rarely use: 1.52 (95% CI 1.16–1.98), 1.50 (95% CI 1.19–1.87), 1.67 (95% CI 1.33–2.08), and 1.60 (95% CI 1.19–2.15), respectively. In the case of women, hair care products, body cleansers, and cosmetics showed significantly higher ORs for use more than once a week versus rarely use of these products: 1.20 (95% CI 1.01–1.42), 1.28 (95% CI 1.04–1.58), and 1.33 (95% CI 1.08–1.65), respectively. Interestingly, ORs were higher in men than in women.

**Table 3:** Odds ratios (OR) and 95% confidence intervals (CI) relating personal care products usage of high urinary concentration of triclosan (male: ≥ 1.096 μg/g creatinine, female: ≥1.329 μg/g creatinine) compared to low urinary concentration of triclosan

	Male		Female	
Category	Unadjusted	Multivariate adjusted model	Unadjusted	Multivariate adjusted model
Fragrance products				
Rarely	1.00	1.00	1.00	1.00
< Once a week	1.55 (1.04–2.29)	1.39 (0.93–2.07)	1.51 (1.15–1.97)	1.27 (0.97–1.67)
≥ Once a week	1.84 (1.43–2.38)	1.52 (1.16–1.98)	1.33 (1.09–1.63)	1.05 (0.84–1.30)
Hair care products				
Rarely	1.00	1.00	1.00	1.00
< Once a week	1.39 (1.03–1.86)	1.39 (1.03–1.87)	1.08 (0.86–1.36)	1.21 (0.96–1.54)
≥ Once a week	1.75 (1.41–2.17)	1.50 (1.19–1.87)	1.19 (1.01–1.41)	1.20 (1.01–1.42)
Body cleansers				
Rarely	1.00	1.00	1.00	1.00
< Once a week	1.45 (0.76–2.78)	1.20 (0.62–2.32)	1.33 (0.73–2.41)	1.20 (0.66–2.20)
≥ Once a week	1.95 (1.61–2.34)	1.67 (1.33–2.08)	1.66 (1.39–1.99)	1.28 (1.04–1.58)
Cosmetics				
Rarely	1.00	1.00	1.00	1.00
< Once a week	1.43 (0.88–2.31)	1.21 (0.74–1.98)	1.12 (0.74–1.70)	1.04 (0.68–1.58)
≥ Once a week	1.84 (1.39–2.45)	1.60 (1.19–2.15)	1.63 (1.33–1.99)	1.33 (1.08–1.65)
Nail care products				
Rarely	1.00	1.00	1.00	1.00
< Once a week	0.99 (0.20–4.93)	1.06 (0.21–5.30)	1.40 (1.16–1.69)	1.26 (1.04–1.53)
≥ Once a week	–	–	1.27 (0.97–1.67)	1.13 (0.85–1.50)
Antimicrobial agents				
Rarely	1.00	1.00	1.00	1.00
< Once a week	1.58 (0.91–2.77)	1.43 (0.81–2.52)	1.17 (0.81–1.68)	0.98 (0.67–1.42)
≥ Once a week	1.47 (1.14–1.90)	1.27 (0.98–1.65)	1.37 (1.12–1.69)	1.17 (0.95–1.44)
Air fresheners				
Rarely	1.00	1.00	1.00	1.00
< Once a week	0.85 (0.49–1.50)	0.82 (0.47–1.46)	1.25 (0.87–1.79)	1.20 (0.84–1.72)
≥ Once a week	1.10 (0.89–1.35)	0.96 (0.78–1.20)	1.17 (0.98–1.41)	1.03 (0.85–1.24)

Multivariate adjusted model: adjusted with age, BMI, education, marital status, household income, alcohol intake, exercise, and smoking

## Discussion

In this study, general characteristics for high urinary TCS concentration were younger age, higher educational level, higher household income level, drinkers, and vigorous exercise group. In a previous study in Canada, higher urinary TCS concentrations were found among women with higher education and income levels, with higher TCS exposure levels in those over 25 years of age than in those under age 25 years [19]. In the United States (US), a study among the general population showed that the higher the income, the higher the concentration of urinary TCS, in relation to age, with the highest concentrations among participants in their 30s; concentrations then gradually decreased with age [2]. On the other hand, according to a study conducted in Korea in 2009, older age and lower income levels were associated with higher urinary TCS concentration with no significant difference [20]. Explanations for the differing results among studies may be owing to differences in lifestyles, such as different frequency of use of TCS-containing consumer products; additional research is needed to clarify these differences in study findings [20]. There has been no confirmation of the common trend found among studies regarding characteristics of drinking, smoking, exercise, and BMI of participants. The high TCS exposure among young adults aged 20–30 years is important in that it can be associated with pregnancy and childbirth. TCS is an endocrine disruptor and several animal studies have found that it can affect thyroid hormone homeostasis [1]. Because thyroid hormones are essential for development of the fetal nervous system and differentiation of the brain, deficiency of thyroid hormones can cause fetal neurodevelopmental problems [21–23].

According to analysis of the relationship between the frequency of use of personal care products and urinary TCS concentration, we found that the frequency of use of hair care products, body cleansers, and cosmetics were consistently related to urinary TCS concentration in both men and women. In the upper 75th percentile of urinary TCS concentration, the proportion of more frequent products use was higher than among participants with rarely products use and ORs were higher in participants who used these products more often. Similar results were found in a study of breastfeeding mothers in Sweden. Mothers who used personal care products containing TCS showed significantly higher breast milk and plasma TCS concentrations than those who did not use these products [8]. In addition, men showed higher ORs than women. Men tend to use less personal care products compared to women, which may be the cause of the larger differences between used and unused groups. These results suggest that personal care products are a major source of TCS exposure.

As a result of analyzing a list of the raw materials of cosmetics reported to the Korea Food & Drug Administration (KFDA) by domestic manufacturers in 2015, 127 of the 100,190 (0.13%) cosmetics products manufactured in Korea were found to have used TCS [24]. It has been found that TCS is mainly used in products that are rinsed off after use, such as body cleansers and foam cleansers [24]. According to US data in 2010, 28 of 3359 eye make-up products, 10 of 4345 facial make-up products, 226 of 3070 body cleansing products, 3 of 5242 hair care products, and 42 of 2842 fragrance products contained TCS [25]. TCS is mainly found in body cleansing products and it is thought that exposure via these products is high. A smaller number of cosmetics and hair care products contain TCS, but these can result in direct exposure and TCS-containing cleansing products are often used to remove cosmetics. Therefore, urine TCS concentrations may be high depending on the use frequency of cosmetics and hair care products.

According to the results of previous studies both on TCS toxicity in humans and use of personal care products as the main exposure source of TCS, regulations on the use of TCS for personal care products have been established in many countries. In Korea, KFDA launched a new regulation with TCS concentration in personal care products, such as, body cleansing products that are rinsed off after use, deodorants (excluding spray products), face powder, and foundation like concealer, that the concentration limit of TCS in personal care products was 0.3%; moreover, TCS could not be used in toothpaste and mouthwash in 2015 [24]. In Europe, the concentration limit of TCS in mouthwash was 0.2% and that in other personal hygiene products was 0.3%. In the case of Japan, the concentration limit of TCS in all products 0.1% [24]. Although TCS regulation has started in many countries, some cosmetics and human body cleansing products still contain TCS and these products still can be purchased in countries with no regulations in place. Regulatory concentrations are set at levels that are harmless to the human body [24]; however, women of childbearing age, children, and adolescents should select products that do not contain TCS or should rinse them off thoroughly after use. It is especially important to be careful when purchasing mouthwash in some foreign countries because nearly 100% of TCS can be absorbed in the oral cavity [3].

In the present study, similar to those of other countries, we found a tendency for urinary TCS concentration to increase in younger age, higher income level and education level. The present study findings also confirmed that personal care products represent the main cause of TCS exposure. People with higher levels of income and education tend to have greater interest in personal hygiene and tend to purchase personal care products, which may lead to increased exposure to chemicals such as TCS. Likewise, young people in their 20s and 30s are more concerned about personal care than children, adolescents, and elderly adults; therefore, this young population is more exposed to TCS through greater use of personal care products. TCS in women of childbearing age can be a potential neurotoxic agent for the fetus during pregnancy [26] and can be delivered to infants through breast milk. Infants and young children are highly susceptible to toxic substances as they have rapid rates of body organ development and their processes of detoxification of toxic substances are less developed [27]. Therefore, additional research is needed to determine whether current levels of regulation are appropriate for these susceptible population.

The limitations of this study are as follows. First, we did not identify the usage amount of personal care products, only the frequency of use. Second, we did not consider the simultaneous use of other personal care products, use of other personal care products not included in the survey, and other TCS exposure factors apart from personal care products. Third, in about 60% of participants, urinary TCS concentration was measured below the detection limit. However, it did not affect the statistical analysis of this study. Fourth, the health effects of increased urinary TCS concentration were not analyzed. Despite these limitations, this study suggested the exposure level of TCS and the relationship with use of personal care products, which are major exposure sources of TCS using a representative data in Korean population.

Regulation of TCS was initiated in Korea in 2015 and it is expected that the effect of reduction of TCS exposure by the regulation will be confirmed through the analysis of third KoNEHS data, which was conducted in 2015–2017 and not publically released yet. Furthermore, children over three years old were included in the study population in the third KoNEHS, therefore, the TCS exposure level and health effects could be studied in the susceptible population. Based on future studies, it could confirm the effect of the regulation and suggest another more powerful policy to minimize TCS exposure in Korean population.

## Conclusion

This study found out the association of the use of personal care products and the concentration of urinary TCS in general population in Korea. According to this study, the levels of urinary TCS were higher in female, younger people, and higher-income people. Because TCS is a well-known endocrine disruptor and several health problems related with exposure to TCS have suggested, further studies are needed to monitor the trend of urinary TCS level and explore other health effects with exposure to TCS in general population in Korea using a representative data for general population.

## Additional file

Additional file 1: Medians (interquartile range) of Urinary TCS concentration according to age. The urinary TCS concentration tended to decrease as age increased. (XLSX 11 kb)

## Abbreviations

BMI: Body mass index
CI: Confidence interval
KoNEHS: Korean National Environmental Health Survey
OR: Odds ratio
TCS: Triclosan
